# QuickLib, a method for building fully synthetic plasmid libraries by seamless cloning of degenerate oligonucleotides

**DOI:** 10.1371/journal.pone.0175146

**Published:** 2017-04-13

**Authors:** Pierre Galka, Elisabeth Jamez, Gilles Joachim, Patrice Soumillion

**Affiliations:** Institut des Sciences de la Vie, Université catholique de Louvain, Louvain-la-Neuve, Belgium; Imperial College London, UNITED KINGDOM

## Abstract

Incorporation of synthetic degenerate oligonucleotides into plasmids for building highly diverse genetic libraries requires efficient and quantitative DNA manipulation. We present a fast and seamless method for generating libraries of PCR-synthesized plasmids designed with a degenerate sequence and short overlapping ends. Our method called QuickLib should find many applications in synthetic biology; as an example, we easily prepared genetic libraries of *Escherichia coli* expressing billions of different backbone cyclic peptides.

## Introduction

Many molecular engineering projects rely on the screening of genetic libraries where diversity is generated by a specific method such as error prone PCR, DNA shuffling or incorporation of synthetic and degenerate oligonucleotides. When building such a library, reaching a high diversity is usually an important objective that requires large amounts of properly assembled DNA and highly efficient downstream transformations. This usually involves multiple DNA manipulation steps, which sometimes necessitate laborious and time-consuming optimization.

In the last decade, new cloning strategies have been elaborated for better controlling and facilitating complex in vitro assembly of long DNA sequences. Notably, in 2009, Daniel Gibson and colleagues developed an isothermal method for the easy and seamless assembly of multiple DNA fragments sharing at least 40 bp of terminal overlapping sequences [1]. Widely used by the community of molecular biologists, this in vitro recombination method takes advantage of three enzymatic activities. A 5’ exonuclease first generates ssDNA 3’ overhangs allowing overlapping sequences to anneal with each other. Gaps are then filled and strands are covalently sealed by the subsequent actions of a polymerase and a ligase. Such intermolecular isothermal assembly of DNA fragments was notably combined with QuickChange-type primers for incorporating multiple site-directed mutations into plasmids [2].

However, for building genetic libraries, the construction of billions of transformants may be necessary and there are no routine protocols for that. Here, we modified the Gibson assembly method for creating oligonucleotide-based genetic libraries instead of assembling monoclonal DNA fragments. Combined with full-plasmid PCR synthesis using a long-degenerate and short-non-degenerate pair of primers, the method was adapted into a polyclonal and intramolecular format enabling the construction of highly diverse and seamless genetic libraries of synthetic plasmids featuring a degenerate region.

## Materials and methods

### Enzymes and oligonucleotides

Oligonucleotides were purchased from Eurogentec (Liège, Belgium). Short oligonucleotides were ordered as desalted grade (selective precipitation optimized process) while long degenerate ones were purified by polyacrylamide electrophoresis. Sequences are given in S1 Table. Phusion DNA polymerase, DpnI, T5 exonuclease, and Taq DNA ligase were purchased from New England Biolabs.

### Plasmid preparation

Fresh plasmid DNA was prepared using Sigma Genelute^™^ Plasmid Miniprep kit, from over-night monoclonal culture of the transformed *E*. *coli* strain in 5ml of liquid Lysogeny Broth (LB) containing the appropriate antibiotics at 37°. The final DNA elution was performed with ultrapure sterile water and immediately used for PCR amplifications.

### PCR-synthesis of linear plasmid libraries

The quality of the plasmid matrix is determinant for efficient full-length PCR amplification. Freshly prepared DNA was used in all described experiments, avoiding, if possible, freezing/thawing cycles. The synthesis of plasmid libraries was performed in 50 μl total volume of mix containing: 1x Go-Taq polymerase Flexi Buffer from Promega (no matter which polymerase was used), 4mM of MgCl_2_, 0.5 mM of each dNTPs, 0.5 μM of the forward degenerate primer, 1 μM of the reverse primer, 75 pM of the plasmid matrix and 1 unit of Phusion DNA polymerase. The following PCR protocol was applied: initial denaturation at 95 C for 4min, followed by 20 cycles of denaturation (95C, 90s), annealing (55 C, 30 s) and elongation (72 C, 7 min), and ending by a final elongation (72 C, 10 min). The number of cycles was reduced to 15 for bigger plasmids (>8kb). In general, the number of cycles should not be pushed too high to avoid decreasing the dNTPs/primers concentrations to the point where self-priming of the combinatorial library sequences becomes significant and lead to DNA aggregates.

### Circularization of plasmid library and matrix purge

On ice, 50 μl of crude PCR product (around 5 μg of linear DNA) were added to 150 μl of the following reaction mix: 100mM Tris-HCl pH 7.5, 10mM MgCl_2_, 0.2mM of each four dNTPs, 1mM NAD^+^, 15%(w/v) PEG-8000, T5 exonuclease (2 U/ml), Phusion DNA polymerase (33 U/ml); Taq DNA ligase (1666 U/ml), and DpnI endonuclease (50 U/ml). After mixing, the solution was directly incubated 1 hour at exactly 50 C.

### Electroporation

Before electroporation, 20 μl of the DNA solution was placed on a Millipore VSWP02500 membrane (25 mm diameter, 0.025 μm porosity) floating on a Petri dish filled with 40 ml of ultrapure sterile water. After 1 hour of dialysis, the droplet is recovered by pipetting. 1 μl of dialyzed DNA was mixed with 50μl electro-competent *E*. *coli* Top 10 cells in pre-chilled 1 mm electrodes distance Gene Pulser cuvette (Biorad) and the electroporation was then performed with Gene Pulser Xcell device (voltage 1800V, capacitance 25μF, resistance 200 ohm). Electroporated cells were recovered with 1ml of LB and incubated at 37 C with orbital shaking during 45 minutes before plating on LB plates containing the appropriate antibiotics. The number of transformants is estimated by plating serial 10-fold dilutions and counting colony forming units (cfu) after overnight incubation at 37 C. Typically, between 10^6^ and 10^7^ cfu were obtained per electroporation. Alternatively, the DNA was concentrated at least 10-fold using the “DNA Clean & Concentrator^™^-5” kit from Zymo Research and up to 10^8^ cfu can be obtained with 5 μl in a single electroporation.

## Results and discussion

**Fig 1:**
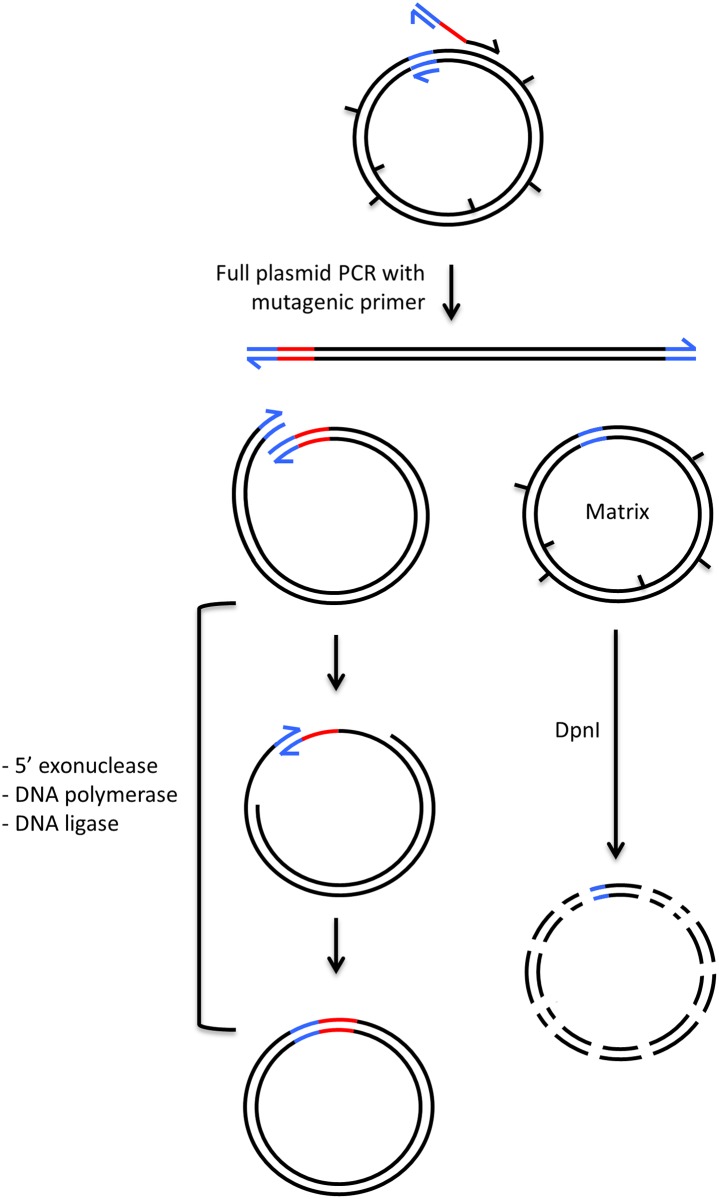
Scheme of the QuickLib method for cloning degenerate oligonucleotides into plasmids. A plasmid is initially PCR-amplified using an asymmetric pair of primers sharing complementary 5’ ends (blue). The randomized sequence in the long primer is coloured in red. The library of linearized synthetic plasmids is then circularized by the combined actions of 3 enzymes and the methylated starting matrix is selectively removed by digestion with DpnI.

The overall method named QuickLib is schematized on Fig 1. It starts with a full plasmid PCR amplification using a long partially degenerate primer and a short non-degenerate primer sharing complementary 5’ ends. Although this reaction is very similar to a QuickChange protocol, the asymmetric nature of the primer pair affords a true exponential amplification rather than a simple replication (see S1 Fig for a detailed mechanism). After the PCR, a Gibson reaction circularizes the library of linear plasmids. Furthermore, the original matrix is eliminated by restriction with DpnI [3], which cleaves exclusively the methylated matrix DNA while synthetic plasmids remain intact.

High yields of full plasmid amplification were obtained with a pair of primers of different lengths. The long primer contains three parts: the 5’ end, of at least 20-nucleotides, is complementary to the small primer; the central part is the degenerate sequence and the 3’ end is the matrix hybridizing sequence. The short primer is the reverse matrix hybridizing sequence and its 5’ end is complementary to the 5’ end of the long primer. Compared to the 40-bp homology required for intermolecular Gibson assembly, the length of the overlapping 5’ ends can be reduced because the intramolecular hybridization required for the subsequent circularization is entropically favored. Typically, between 25 and 30-bp terminal homology resulted in high assembling efficiency. An example of primer sequences and their priming and homology regions is presented in S1 Table. The short primer is introduced in at least two- to four-fold stoichiometric excess compared to the long one so that their mutual pairing does not impair priming at each PCR cycle (S1 Fig). This is contrary to protocols with equal length/equal amount of primers such as with QuickChange mutagenesis. Since some DNA polymerases such as the Taq polymerase have nontemplate-dependent terminal transferase activity adding a deoxyadenosine to the 3’ ends [4, 5], an activity that could impair the next circularization step, the 5’ end of all our primers is designed so that primers start upstream of a deoxythymidine in the target matrix. We called this the AFT priming rule (for AFter T). With these designed primers, full plasmid amplification is performed using classical PCR protocols with any DNA polymerase. Although different polymerases were successfully tested, giving similar numbers of transformants, the cleanest reactions were obtained with proofreading DNA polymerases such as Phusion and with freshly prepared plasmid as matrix. Typically, amplification factors around 4,000-fold were obtained after 20 cycles of PCR, which is equivalent to 12 fully exponential cycles (2^12^ = 4,096).

**Fig 2:**
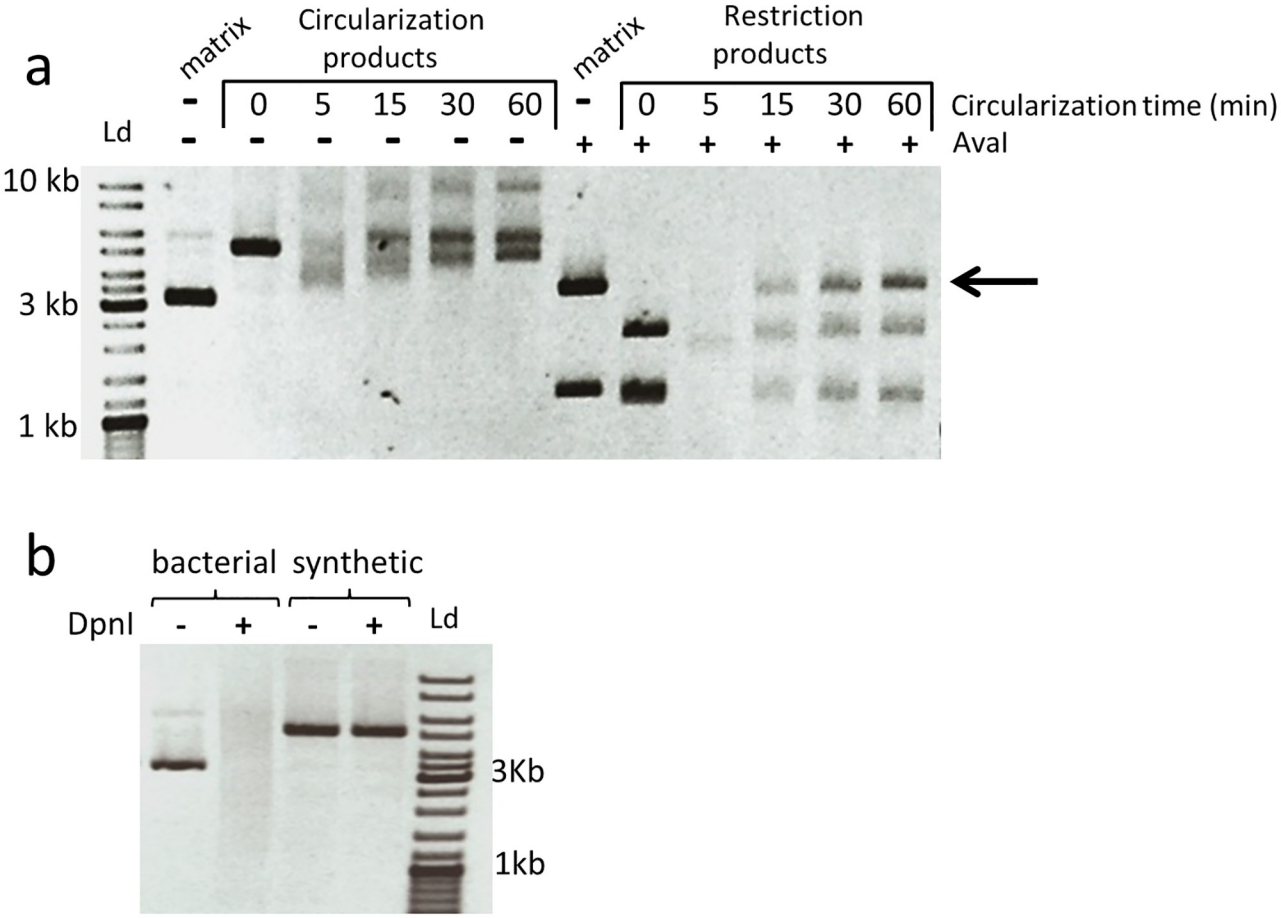
Circularization of linear plasmid library and removal of starting matrix. (a) Time course of the circularization reaction: a mix of four enzymes (T5 exonuclease, DNA polymerase, DNA ligase and DpnI) was added to the amplified linear plasmids and incubated for one hour at 50 C. The amount of T5 exonuclease was reduced 4-fold compared to Gibson’s protocol. Circularization products (left side) are also analyzed by restriction with AvaI (right side). The band at 3.6 kb (black arrow) is only present when the plasmids are sealed. (b) DpnI is cleaving the starting matrix at 50 C while the synthetic PCR product is resistant to its action.

The circularization reaction was then performed directly on the PCR product by adding an adapted mixture of enzymes. We found that better circularization yields were obtained if the amount of exonuclease was reduced by 4- to 10-fold compared to the amounts used by Gibson when assembling multiple DNA fragments. A time-course experiment indicates that, under these conditions, about 50% of a 5kb linear degenerate plasmid (crude PCR product) with 27-bp terminal homology regions could be circularized (Fig 2a, right). Properly assembled DNA is usually not detectable on agarose gels when performing classical protocols such as QuickChange or two-fragments Gibson cloning. This experiment also shows that the exonuclease is active at 50°C during the first minutes of incubation after adding the mixtures of enzymes (Fig 2a, left). It is then inactivated allowing the polymerase to fill the gaps and the ligase to seal the DNA molecules. Since the circularized synthetic plasmids are no longer substrates for any enzymes present in the mixture, so the global reaction of this circularization process evolves spontaneously in the direction of the product.

About 50% of the PCR products are not properly circularized as shown by the two smaller bands that are generated upon restriction with AvaI (Fig 2a, right). This probably originates from the hybridation between the forward and reverse primers that prevents one of the homology regions to be fully replicated during the PCR reaction. The circularization is not possible without both 3’ homologous extremities, (Fig 1). Instead, such incomplete fragments may assemble intermolecularly and generate linear dimers of plasmid as suggested by the band appearing at 10 kb after long reaction time (Fig 2a, left). This hypothesis is schematized in S2 Fig. However, these byproducts are not considered as a problem since they are not able to transform bacteria. Sequencing 350 individual clones did not reveal a single clone with a double sequence in the degenerate region, which would have been indicative of a concatenate plasmid. Moreover, this also indicates that the observed 10 kb fragment does not contain significant amounts of circularized products, meaning that circularization is essentially intramolecular.

The presence of wild-type plasmids (estimated at around 10^6^ molecules per μl in the final product) can generate a strong unwanted background in the genetic libraries. Treating the circularization product with DpnI, an endonuclease that cleaves selectively methylated G^m^ATC sites, easily eliminates this background. As shown in Fig 2b, DpnI conserves its activity at 50°C in the circularization buffer conditions and can be directly added to the enzyme mix, therefore simplifying the experimental procedure. Using this circularization/purge step, the wild-type background was successfully decreased, reducing the matrix type clone frequency to less than 1% in four independent experiments since only one parental clone was identified upon sequencing 350 individual clones. Moreover, the addition of this fourth enzyme did not affect the circularization efficiency.

**Fig 3:**
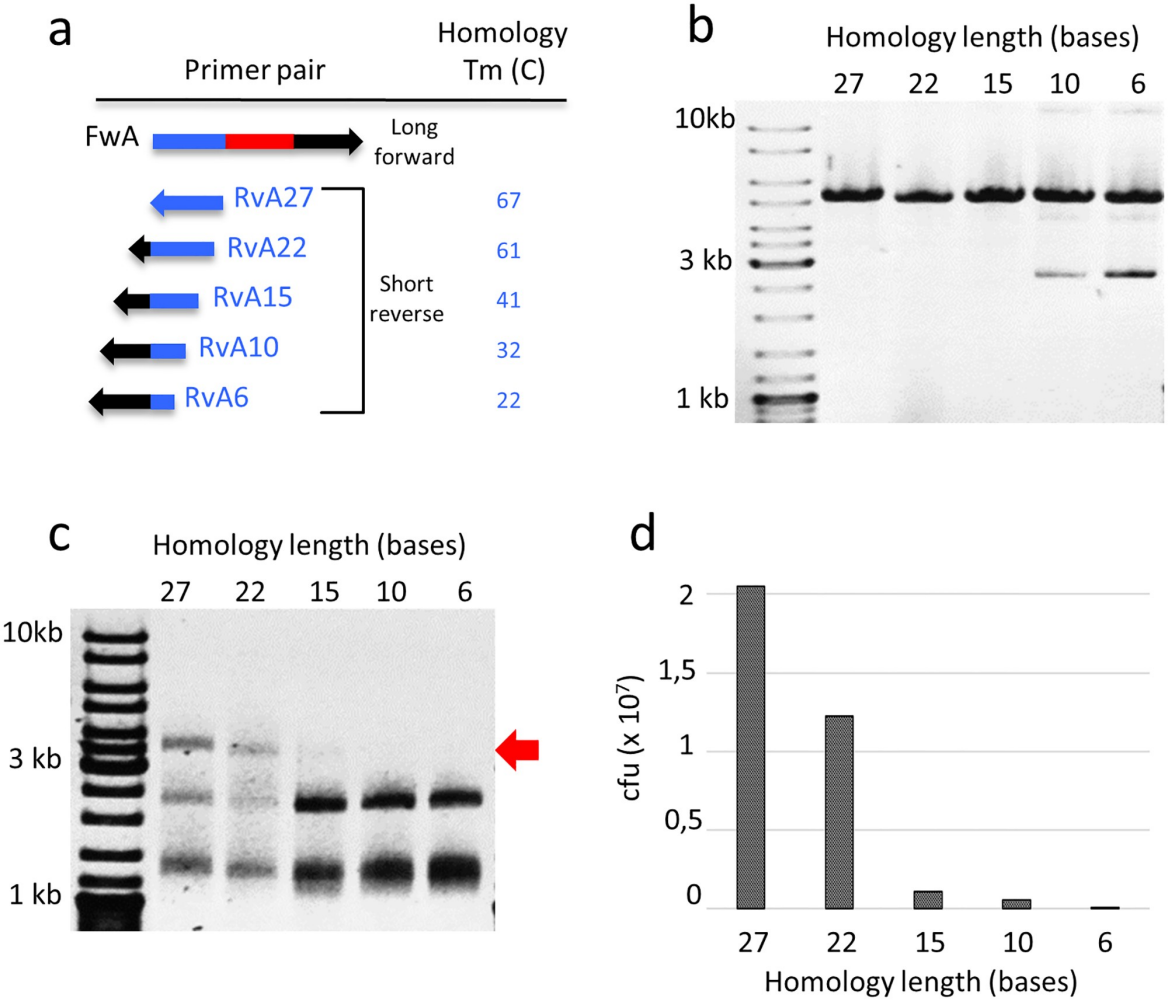
Dependence of 5’ homology length of primer pairs on the efficiency of QuickLib assembly. (a) A long degenerate primer (84 nt) and different short primers (27 nt) sharing the same overall length but with variable homology length were tested as primer pairs (full sequences are given in S1 Table). The melting temperatures of duplexes between forward and reverse primers, i.e. between the homology regions that must be annealed for the circularization step, are listed. (b) Full plasmid PCRs gave similar yields of linear plasmids. (c) After circularization/purge reaction, the amount of circularized product (red arrow) was estimated by AvaI restriction. (d) Overall efficiency was evaluated by measuring the number of colony forming units (cfu).

The minimal homology length required for efficient assembly was also evaluated. Short primers were designed with identical lengths but variable homology region to the long primer (Fig 3a). Full plasmid PCR were equivalently efficient (Fig 3b) while the amount of circularization products was dependent on the homology length (Fig 3c) and correlated with the number of obtained transformants (Fig 3d). A sharp 10-fold drop in transformation yield is observed when the homology region is shortened from 22 to 15 nucleotides. Not surprisingly, this corresponds to the point where the melting temperature of the homology region goes below the temperature of the circularization reaction (50 C). Homology regions longer than 27 nt were not tested because the yield of circularization was already high (around 30 to 50%) and because it would have required a longer degenerate primer which was already quite long (84 nt). Moreover, the melting temperature between the long and short primers was kept below the polymerization temperature of the PCR reaction (72 C) for minimizing the production of fragments that cannot be circularized (S2 Fig).

We applied our method for building libraries of biosynthetic cyclic peptides based on the splicing of a permuted intein [6]. This requires the seamless cloning of a degenerate oligonucleotide inside the open reading frame encoding a permuted intein. A long primer containing a central part of eight successive NNB codons (B = G/T/C) was successfully used for preparing approximately 2.5 μg of degenerate and circularized plasmids in a total volume of 200 μl. After 1-hour dialysis on a floating membrane, 1 μl was sufficient for obtaining between 10^6^ and 10^7^ individual transformants using commercial electrocompetent *E*. *coli* (TOP10). Up to 10^8^ transformants per electroporation were obtained with concentrated DNA. Sequencing 200 transformants picked at random revealed the expected sequence for 198 of them. Two sequences contained additional inserted bases resulting in frameshifts. The method was also successfully used for several other mutageneses with similarly designed primers (S1 Table) and plasmid matrices ranging from 5 to 9 kb. In all cases, similar numbers of transformants were obtained. The maximal length that can be degenerate is limited by the capacity to synthesize long oligonucleotides and is around 60 nucleotides. Potentially, two vicinal sequences can be randomized simultaneously if a second long degenerate primer is used instead of the short one, Noteworthy, the method is also well appropriate for difficult site directed mutageneses such as when performing multiple substitutions, insertions or deletions.

In comparison with other protocols that have been used for cloning degenerate oligonucleotides, our one-day method is much simpler and much faster. For example, small synthetic double stranded DNA cassettes have been prepared from degenerate oligonucleotides and cloned by restriction and ligation [7]. Library construction took several weeks, notably because the plasmid must be initially mutated for installing the appropriate restriction sequences at the cloning site. Alternatively, degenerate oligonucleotides can be used as PCR primers, similarly to QuickLib, but intermolecular cloning by restriction ligation was then performed, requiring several additional steps and at least four days [8,9]. Moreover, all these methods necessitated difficult restriction of small synthetic DNA or PCR fragments. Restriction enzymes recognizing non-palindromic sequences have also to be used if the final construct must not contain any scar [7,9].

In conclusion, we present a powerful, fast and simple method for the site specific and seamless incorporation of degenerate oligonucleotides into plasmids. The seamless cloning strategy should facilitate the genetic constructions of ready-to-screen libraries and should find a wide range of applications in directed evolution and synthetic biology projects. Notably, the method is particularly well suited when precise randomizing of a defined amino acid or nucleotide sequence within a protein or DNA/RNA element is required.

## Supporting information

S1 Fig Scheme of the full-plasmid PCR. (PDF)

S2 Fig Impaired circularization of some PCR products. (PDF)

S1 Table List of oligonucleotides. (DOCX)
