# Supplementary material for: QuickLib, a method for building fully synthetic plasmid libraries by seamless cloning of degenerate oligonucleotides
Source: PLoS One. 2017 Apr 13;12(4):e0175146. doi: 10.1371/journal.pone.0175146 (PMC5390991; doi:10.1371/journal.pone.0175146)
Supplement: S1 Fig — (PDF) [file pone.0175146.s001.pdf]

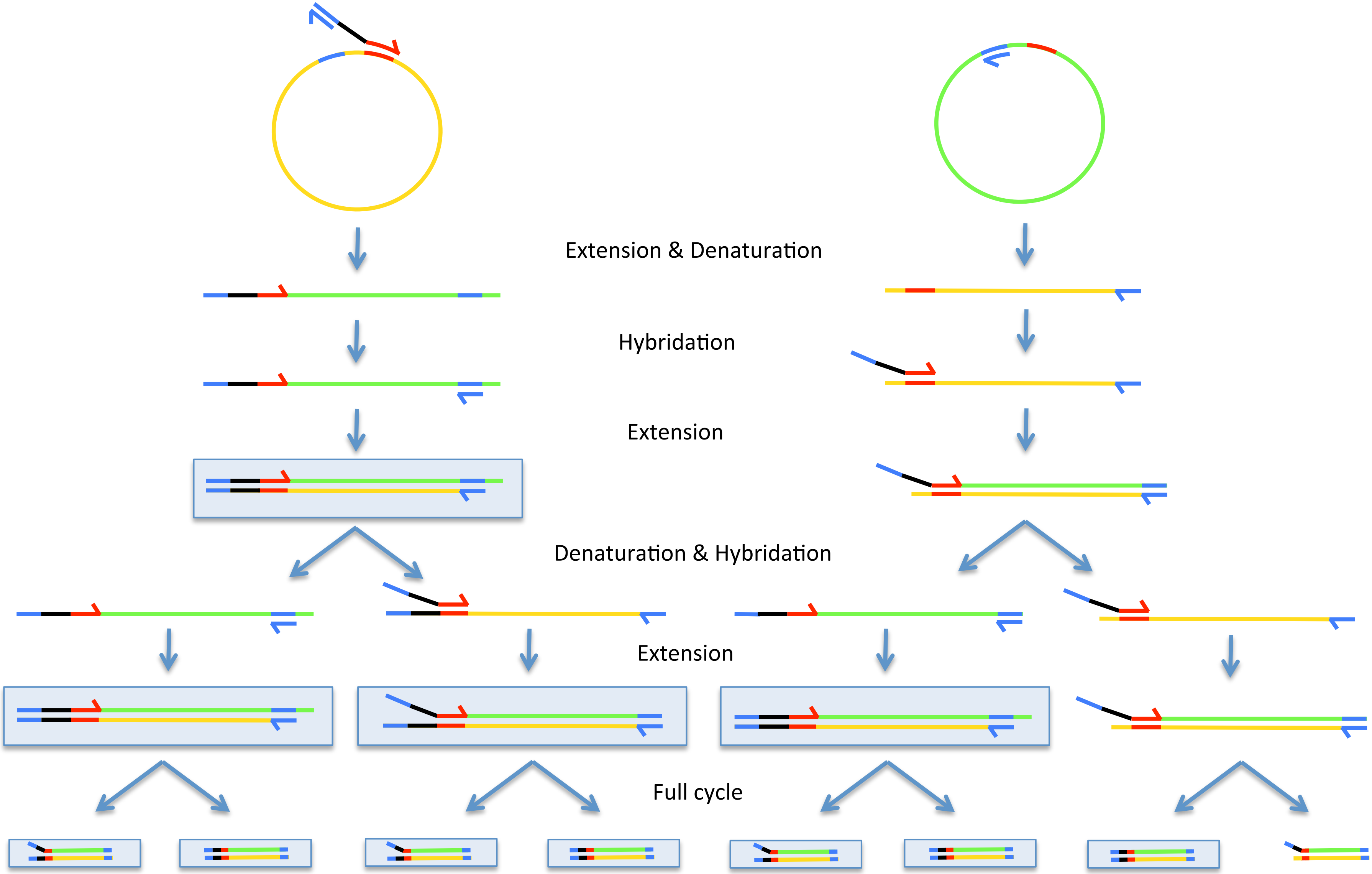

S1 Fig. Scheme of the full-plasmid PCR. In red and blue are the vicinal hybridation regions on the matrix, PCR products and pair of primers. The degenerate region is coloured in black and prevents complete base pairing upon hybridation. Both complementary strands of the plasmid are represented in orange and green, and are initially separated for clarity. The PCR products that can be further circularized by adding the Gibson mix of enzymes are boxed.
