# Supplementary material for: QuickLib, a method for building fully synthetic plasmid libraries by seamless cloning of degenerate oligonucleotides
Source: PLoS One. 2017 Apr 13;12(4):e0175146. doi: 10.1371/journal.pone.0175146 (PMC5390991; doi:10.1371/journal.pone.0175146)
Supplement: S2 Fig — (PDF) [file pone.0175146.s002.pdf]

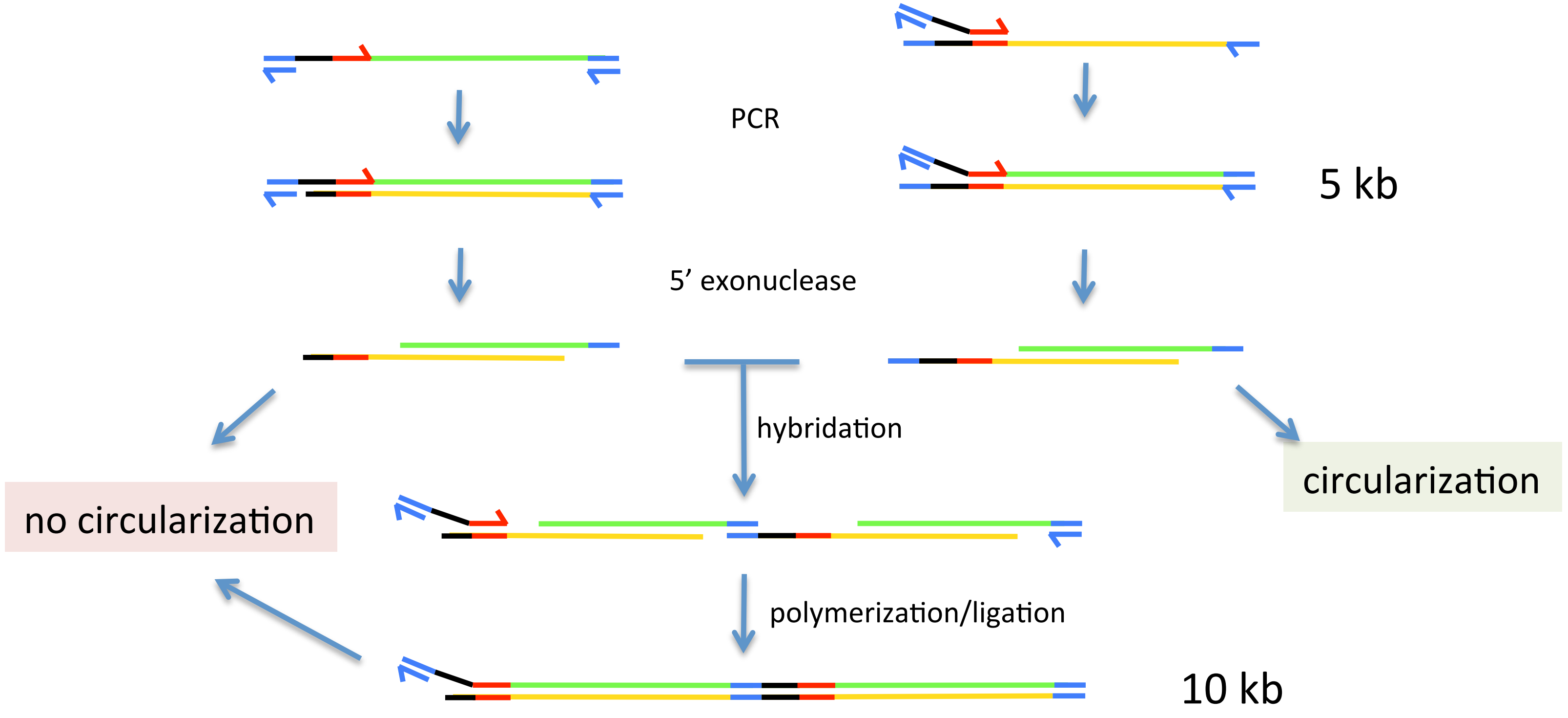

S2 Fig. Impaired circularization of some PCR products. In blue are the homology regions and the priming site of the small primer. The degenerate region is coloured in black and the priming site of the degenrate primer is in red. Both complementary strands of the plasmid are represented in orange and green, and are initially separated for clarity. If a small primer (in excess) hybridize to the long one, the PCR elongation of one of the strands (top left) may not proceed up to the end and the homology region on the side of the long primer won’t be replicated, thus preventing the subsequent circularization. This fragment may however assemble with another one for creating a linear dimer of plasmids that cannot be circularized as well (bottom).
