# Supplementary material for: QuickLib, a method for building fully synthetic plasmid libraries by seamless cloning of degenerate oligonucleotides
Source: PLoS One. 2017 Apr 13;12(4):e0175146. doi: 10.1371/journal.pone.0175146 (PMC5390991; doi:10.1371/journal.pone.0175146)
Supplement: S1 Table — (DOCX) [file pone.0175146.s003.docx]

**QuickLib, a method for building fully synthetic plasmid libraries by seamless cloning of degenerate oligonucleotides**

Pierre Galka, Elisabeth Jamez, Gilles Joachim and Patrice Soumillion

S1 Table. Oligonucleotides Used for Building Libraries with QuickLib Protocol

| Name | Sequence (5’->3’)^a,b^ | Purpose |
| --- | --- | --- |
| FwA | GTCGCCAATGACATCATTGTCCATAACAGT*NNBNNBNNBNNBNNBNNBNNBNNB*GGATGCATCAGTGGAGATAGTTTGATCAGC | Library of cyclic decapeptides with eight degenerated positions (NNB codons) |
| FwA2 | GTCGCCAATGACATCATTGTCCATAACAGT*NNBNNBNNBNNBNNBNNBNNBNNBNNYNNY*GGATGCATCAGTGGAGATAGTTTGACAGC | Library of cyclic dodecapeptides with ten degenerated positions (NNB and NNY codons) |
| FwA3 | GTCGCCAATGACATCATTGTCCATAACAGT*NNYNNYNNYNNYNNYNNYNNYNNYNNYNNYNNYNNY*GGATGCATCAGTGGAGATAGTTTGATCAGC | Library of cyclic tetradecapeptides with twelve degenerated positions (NNY codons) |
| FwB | TCAGGAATGGGGTCTAGAAAATACC*SKNN9KRA9MRT988GAG77KGAKNTN66997KRNNNNK77K88KRANNNKMSRR*ACACCACTAGTCCGCGCGAC | Library of enzymes with semi-rational randomization of an active site flanking loop |
| FwC1 | TGTTTTAGATATAACCAGTAAGGCCGAT*MRYCWYMRYMRYMRYMMYCANSMYMRYSAYSWYMWYMMYM*ATCAAAGAAGTATTGCGACA | Library of porins with semi-rational randomization of a gating region |
| FwC2 | TGTTTTAGATATAACCAGTAAGGCCGATAG*NCWYAGNMRYMRYACNCANSMYMRYSAYGTNMWYACNM*ATCAAAGAAGTATTGCGACA | Library of porins with semi-rational randomization of a gating region |
| FwD1 | CAGAAGGGTTCTATTTCTGTTGGCCAG*MAYSWYCMYYWYMWYMMYSRYCRYSWYMMYSRYSANMRYSMYM*ATGTAAATAATCCATTTCAG | Library of porins with semi-rational randomization of a gating region |
| FwD2 | CAGAAGGGTTCTATTTCTGTTGGCCAG*MAYGTNCMYTTYMWYMMYGGNCRYSWYACNSRYSANMRYGCNM*ATGTAAATAATCCATTTCAG | Library of porins with semi-rational randomization of a gating region |
| RvA27 | GTTATGGACAATGATGTCATTGGCGAC | Reverse primer used with FwA, FwA2-3 |
| RvA22 | GGACAATGATGTCATTGGCGACAAAGT | Reverse primer used with FwA |
| RvA15 | GATGTCATTGGCGACAAAGTTATGTGG | Reverse primer used with FwA |
| RvA10 | CATTGGCGACAAAGTTATGTGGTCCTG | Reverse primer used with FwA |
| RvA6 | GGCGACAAAGTTATGTGGTCCTGGCAC | Reverse primer used with FwA |
| RvB | GGTATTTTCTAGACCCCATTCCTGA | Reverse primer used with FwB |
| RvC | CGGCCTTACTGGTTATATCTAAAACAA | Reverse primer used with FwC1-2 |
| RvD | GGCCAACAGAAATAGAACCCTTCTGAC | Reverse primer used with FwD1-2 |

^a^Underlined : complementary 5’ ends ; italicized : degenerated region

^b^R=(A,G) ; S=(G,C) ; K=(G,T) ; M=(A,C) ; Y=(C,T) ; W=(A,T) ; B=(T,C,G) ; D= (A,T,G) ; H=(A,T,C) ; V=(G,A,C) ; N=(A,T,G,C) ; 6=(90% A, 10% B) ; 7=(90% C, 10% D) ; 8=(90% G, 10% H) ; 9=(90% T, 10% V).
